# Epigenetic Biomarkers: Potential Applications in Gastrointestinal Cancers

**DOI:** 10.1155/2014/464015

**Published:** 2014-03-06

**Authors:** Jiaqiu Li, Hongchuan Jin, Xian Wang

**Affiliations:** Department of Medical Oncology, Sir Runrun Shaw Hospital, Medical School of Zhejiang University, Hangzhou 310016, China

## Abstract

Genetics and epigenetics coregulate the cancer initiation and progression. Epigenetic mechanisms include DNA methylation, histone modification, chromatin remodeling, and noncoding RNAs. Aberrant epigenetic modifications play a fundamental role in the formation of gastrointestinal cancers. Advances in epigenetics offer a better understanding of the carcinogenesis and provide new insights into the discovery of biomarkers for diagnosis, and prognosis prediction of human cancers. This review aims to overview the epigenetic aberrance and the clinical applications as biomarkers in gastrointestinal cancers mainly gastric cancer and colorectal cancer.

## 1. Introduction

Cancer is one of the major disorders threatening our life. Gastrointestinal cancers mainly, including gastric cancer (GC) and colorectal cancer (CRC), account for a large proportion of human malignancies. They are both aggressive and the common cause of cancer-related deaths with a high disease-specific mortality rate around the world. There have been a great number of studies on the pathogenesis of gastrointestinal cancers. With a long history of chronic inflammation, GC and CRC result from the accumulation of both genetic and epigenetic changes that cause the transformation of normal cells into cancer cells. The classic genetic alterations are the mutations in key tumor suppressor genes or oncogenes, leading to defects of protein functions or deregulation of gene expression. In contrast, epigenetic events could affect gene expression without any changes in DNA sequence.

## 2. Overview of the Epigenetics

The term epigenetics was coined in 1942 by C. H. Waddington when he was studying the causality between the genotype and the phenotype [1]. Now epigenetics refers to heritable modifications of the genome without any changes in primary DNA sequences [2]. In 1982, Feinberg and Vogelstein first discovered aberrant epigenetic alterations in human colorectal cancer [3]. Epigenetics which focuses on the process transforming genotype into phenotype is corresponding to genetics that refers to the heredity of genotype. Epigenetic alterations, like gene mutations, contribute to the pathogenesis and molecular heterogeneity of cancers. Epigenetics is different from the traditional genetics, mainly in the reversibility and position effect. The epigenetic modifications currently believed to play a role in cancers include DNA methylation, specific histone modifications, chromatin remodeling, and noncoding RNAs.

### 2.1. DNA Methylation

The best-characterized epigenetic modification is methylation, a covalent addition of a methyl group to cytosines within CG dinucleotides by DNA methyltransferases (DNMTs) [4]. CG dinucleotide sequence, termed as CpG, is the favored substrate for the DNMTs in mammalian cells. The genome CpG islands are regions where the percentage of the CpG dinucleotides is higher. Generally, CpG islands are defined as sequences greater than 200–500 bases in length with greater than 50% GC content and a CpG ratio of greater than 0.6 [5]. CpG islands mainly exist in the promoter region of genes and are inclined to become aberrantly methylated in cancer cells [6]. Methylation of CpG islands with the promoter region is correlated with transcriptional silencing while methylation that occurs in CpG sites outside of promoter regions, termed as gene body methylation, has been associated with transcriptional activation [7]. In the process of tumor formation, demethylation of the entire genome and hypermethylation in the CpG islands of gene promoters occur simultaneously [8]. A wide range of hypomethylation can cause the change of chromatin structure, lower degree of condensed chromatin, and the increase of genome instability, leading to the occurrence of tumor eventually. For instance, microsatellite DNA sequences are easier to mutate when they are hypomethylated, which have been identified in many kinds of tumor models [9]. On the other hand, the silence of important genes such as tumor suppressor genes due to the hypermethylation in the CpG islands of gene promoter also contributes to tumor developments [10]. Methyl-binding proteins (MBPs) that bind with high affinity to methylated DNA can indirectly block the access of transcription factors to the promoter regions [11]. As mentioned above, the methylation state of genes is regulated by DNA methyltransferases (DNMTs). Among them, DNMT1 is responsible for the maintenance of existing DNA methylation while DNMT3a and DNMT3b catalyze DNA methylation in a de novo fashion [12, 13].

### 2.2. Histone Modification

Another critical epigenetic mechanism refers to the modifications of the histone tails, such as acetylation, methylation, phosphorylation, ubiquitination, and sumoylation [14, 15]. Two subunits of each of the following histone proteins such as H2A, H2B, H3, and H4 form an octamer which is wrapped by DNA to make up a nucleosome, the basic unit of chromatin [16]. Histones are proteins containing a globular domain and a flexible charged NH_2_ terminus known as the histone tails that are prone to undergo posttranslational modifications. The interaction between DNA and histones alters the accessibility of DNA transcriptions sites to RNA polymerase II or other transcription factors. These posttranslational modifications to histone tails govern the structural state of chromatin and the resulting transcriptional status of genes within particular sites [17]. As a well-studied covalent modification, histone acetylation is controlled by histone acetyltransferases (HATs) that add an acetyl group to lysine residues and histone deacetylases (HDACs) responsible for removing the acetyl group. Generally, HATs can promote the transcription by neutralizing a positive charge to cause the chromatin open and subsequent transactivation of specific genes while HDACs lead to chromatin condensation and transcriptional inactivation of the involved DNA [18, 19]. HDACs can be divided into four catalytic groups, referred to as classes I (HDAC 1–3 and 8), II (HDAC 4–7, 9, and 10), III (Sir-2 related-protein 1–7), and IV (HDAC11) [20]. Deregulation of HADC activity has been strongly implicated in aberrant gene silencing and tumorigenesis, providing a molecular rationale for targeting HDACs activity in the clinical intervention of human cancers [21]. Histone methylation is another important way to regulate histone which usually happens on lysine and arginine residues of histones H3 and H4. The methylated histone could realize fine control of cell functions by means of collecting many kinds of DNA regulatory factors. The methylation of histone tails is regulated by histone methyltransferases (HMTs) and histone demethylases (HDMs). In 2004, Shi et al. first confirmed that LSD1 (histone demethylase SWIRM1) could mediate histone demethylation, changing the viewpoint that histone methylation was irreversible [22, 23]. Lysine residues might present different levels of methylation, mono-, di-, and trimethylation, leading to various states of the genome [24, 25]. Depending on the residue and the level of methylation, the chromatin might be open such as trimethylation at H3K4 and H3K36 or closed such as trimethylation at H3K27, H3K9, and H4K20 and dimethylation at H3K9 [26]. In addition, histone phosphorylation will affect chromatin structure. For instance, ERK-MAPK (mitogen-activated protein kinases) pathway can induce H3 S10 phosphorylation to prompt chromatin condensation essential for the progression of mitosis [27]. Taken together, multiple combinations of histone modifications in specific genomic regions could contribute to a more “open” or “closed” chromatin structure resulting in the activation or the repression of gene expression [28].

### 2.3. Chromatin Remodeling

Chromatin remodeling refers to changes of chromatic location and structure and mainly gives rise to the loss of tightened chromatin in nucleosome joint to expose cis-acting elements in the gene promoter and provide the chance of the combination with trans-acting factors [29]. The process of chromatin remodeling is mediated by ATP (adenosine triphosphate) dependent nucleosome remodeling complex and histone covalent modification complex. The former changes the configuration of nucleosome through ATP hydrolysis while the latter catalyzes the covalent modifications on the histone tails. These complexes work in concert with activating chromatin-modifying enzymes that can be categorized into two families, the ISWI family mobilizing nucleosomes along the DNA and the SWI/SNF (SWItch/Sucrose NonFermentable) family that transiently alters the structure of the nucleosome, whereby exposing DNA [30, 31]. Dynamic chromatin remodeling is the basis of many biological processes such as gene transcription, DNA replication, and DNA damage repair. Therefore, the chaos of such biological processes is directly related with the occurrence and development of tumors.

### 2.4. Noncoding RNAs

The RNA world was expanded by the recent identification of regulatory noncoding RNAs (ncRNAs), challenging the long-standing assumption that RNA is an intermediate between stable genes and versatile proteins. Actually, most of the genome in mammals and other eukaryotes is transcribed in a developmentally regulated manner to produce large amount of noncoding RNAs. Depending on the functional or biochemical features, ncRNAs can be divided into long noncoding RNAs (lncRNAs) that are longer than 200 nucleotides and small regulatory RNAs such as microRNAs (miRNAs), short interfering RNAs (siRNAs), piwi-interacting RNAs (piRNAs), small nucleolar RNAs (snoRNAs), and other short RNAs.

The most studied class of ncRNAs is miRNAs that are small ncRNAs of approximately 22 nucleotides and responsible for posttranscriptional gene silencing of more than 60% of protein-coding genes by controlling mRNA translation into proteins [32, 33]. miRNAs play their roles through either cleavage of mRNA or translational repression when pairing with the 3′UTR (untranslated region) region of target genes in an incomplete complementary way [34, 35]. miRNAs are involved in the regulation of many important biological processes, such as cell differentiation, proliferation, and apoptosis [36, 37]. The abnormal expression of miRNAs has been linked to cancers and now is being used to classify many human cancers [38]. The first miRNAs were identified as lin-4 and let-7 in 1993. Both miRNAs have important roles in controlling developmental timing, and when they are inactivated, epithelial cells will go through additional cell division instead of normal differentiation [39]. The transcription of genes coding miRNAs is regulated in a similar manner to the transcription of protein-coding genes [40]. For instance, miRNAs dysregulation can occur through DNA hypermethylation, affecting the production of their primary transcript. Most of the miRNAs are generally downregulated in cancers while a few miRNAs, termed as oncomiRNAs, show elevated expression.

## 3. Epigenetic Biomarkers in Gastric Cancer and Their Applications

### 3.1. DNA Methylation in GC

Gastric cancer (GC) is the fourth most frequently diagnosed cancer and the second leading cause of cancer-related deaths worldwide [41]. Incidence of gastric cancer is affected by geographic, ethnic, and cultural factors in addition to *Helicobacter pylori* infection which always influences the mucosa of the stomach and leads to inflammation [42, 43]. Gastric cancer is a highly heterogeneous disease. Therefore, it is necessary to figure out the alterations involved in individual gastric cancer so as to increase the chance to predict prognosis and establish effective treatment options. Gastric adenocarcinoma accounting for 90–95% of gastric cancers has two histological types—intestinal and diffuse types based on microscopic observation and growth patterns. They are widely different in their molecular pathogeneses [44]. Nonetheless, epigenetic alterations play important role in the development of both types of gastric carcinomas.

DNA methylation mapping in cancer genomes shows that the vast majority of cancer types exist in hundreds of genes with high or low methylation and the highest CpG island hypermethylation frequency takes place in gastric cancer [45, 46]. A number of tumor suppressor genes acting in cell cycle, apoptosis, cell adhesion, and invasion are inactivated by hypermethylation such as CDH1 (cadherin 1) and MLH1 (mutL homolog 1). E-cadherin (CDH1) is a cell-to-cell adhesion protein which exists ubiquitously at adherent junctions of epithelial cells. Inactivations of CDH1 include the loss of heterozygosity (LOH) and DNA hypermethylation of the promoter CpG islands. CDH1 is downregulated in sporadic tumors and associated with a poorly differentiated phenotype and a poor clinical outcome [47, 48]. MLH1, involved in repair of mistakes in replication error (RER) of tandem repeat of the short sequences, is hypermethylated exclusively (80%-100%) in the RER phenotype of GC. Interestingly, MLH1 hypermethylation may be an early event which occurs in precursor cells as the corresponding normal mucosa was also similarly hypermethylated [49, 50]. In addition, HOPX (HOP homeobox) had the highest priority with 84% hypermethylated in GC versus 10% in the corresponding normal tissues [51]. Promoter methylation of PCDH10 (protocadherin 10) was detected in 82% of GC samples compared with 37% in the adjacent nontumor tissues [52]. Its methylation was significantly associated with poor survival in patients with early stage of GC. UCHL1 (ubiquitin carboxyl-terminal esterase L1), responsible for maintaining ubiquitin levels by releasing ubiquitin from tandem conjugated ubiquitin monomers, was commonly silenced through promoter methylation in 77% of GC [53, 54]. Due to the promoter hypermethylation, ADAMTS9 (ADAM metallopeptidase with thrombospondin type 1 motif 9), belonging to the ADAMTS family, was silenced in 75% of GC cell lines [55]. Dkk-3 (dickkopf WNT signaling pathway inhibitor 3), an inhibitor of Wnt signaling, was methylated in 68% of primary GC and it was related significantly and independently with poor survival by multivariate Cox regression analysis [56]. The Kaplan-Meier survival curve revealed that GC patients with methylated Dkk-3 had shorter survival compared with its counterparts—median survival 0.76 years and 2.68 years, respectively. Other relevant candidacy of highly relevant methylation (HRMGs) can be found in the review of Yamashita et al. [57].

Because of the easier availability and detection of methylated DNAs in various body fluids, they can serve as useful noninvasive biomarkers for GC. The detection of specific methylated genes in the blood DNA of GC patients is of potential diagnostic significance, perhaps eventually overriding the value of CEA (carcinoembryonic antigen), a classical tumor marker in the serum. For example, RNF180 (ring finger protein 180) has been shown as a novel preferentially methylated gene in the plasma of GC patients [58]. Promoter methylation of RNF180 was 76% of GC patients with sensitivity 63% and specificity 91%. Overexpression of RNF180 could suppress cell growth and induce apoptosis mediated by upregulating MTSS1 (metastasis suppressor 1), CDKN2A (cyclin-dependent kinase inhibitor 2A), and TIMP3 (TIMP metallopeptidase inhibitor 3). Another preferential methylation in the blood DNA was evident in the genes like SLC19A3 (solute carrier family 19 member 3) [59], MHL1, APC (adenomatosis polyposis coli), TIMP3, and E-cadherin [60]. When combining the use of four methylation markers including MHL1, APC, TIMP3, and E-cadherin, the sensitivity was 55% and the specificity was 86%. Interestingly, aberrant methylation in CpG islands of cancer is not only associated with tumor suppressor genes [61]. The CpG island of hTERT (telomerase reverse transcriptase), coding the catalytic subunit of telomerase, was hypermethylated more frequently in neoplastic than in nonneoplastic gastric mucosa [62]. Whether the methylation of hTERT could be a potential biomarker for GC remains to be clarified.

### 3.2. Histone Modifications in GC

HATs such as p300, CBP, and PCAF (p300/CBP associated factor) have prominent roles in oncogenesis by acetylating multiple histone and nonhistone proteins [63, 64]. Loss of heterozygosity of p300 and missense mutations has been confirmed in gastric cancer [65]. PCAF expression was downregulated in gastric cancer tissues and was correlated with gastric wall invasion, tumor size, and node metastasis stage [66]. On the contrary, patients with high-PACF have a significantly better overall survival. Dysregulation of HDACs activity has also been strongly implicated in abnormal gene silencing and tumorigeneses. Except aberrant gene silencing, altered expression of HDACs such as HDAC1 or HDAC2 has also been observed in gastric carcinoma [67, 68]. The class III HDACs play an important role in cell survival via deacetylation of key cell cycle and apoptosis regulatory molecules including p53 and Rb [69–71]. Histone acetylation has been clinically correlated with pathological epigenetic aberrance in cancers. The reduction of p21 has been validated to be caused by hypoacetylation of histone H3 [72]. By contrast, hyperacetylation in H3 of ZNF312b (FEZ family zinc finger 1) promotes the progression of gastric cancer [73]. Reduced histone H4 acetylation was found in some gastric lesions exhibiting intestinal metaplasia and has been shown to correlate with advanced tumor stage, invasion, and lymph node metastasis in gastric patients [74, 75]. All of these suggest levels of histone acetylation may be closely associated with the development and progression of gastric carcinomas, and the loss of acetylation of specific residues could be as epimarkers of tumor cells [76]. Meanwhile, the methylated levels of H3K9 have been confirmed to be relevant with higher stage, lymph node metastasis, recurrence, and worse prognosis partly due to inactivation of some tumor suppressor genes [77]. Overexpression of phosphorylated histone H3 was related with intestinal type, vessel invasion, lymph node metastasis, and even a poor prognosis in gastric adenocarcinoma [78].

### 3.3. miRNAs in GC

Among various ncRNAs, miRNAs are well studied. It is estimated that up to 30% of genes in the human genome are regulated by miRNAs [79]. Owing to their smaller size, high stability in human tissues, and crucial translational regulatory function, miRNAs have strong potential as better biomarkers than mRNA and proteins [80]. A direct link between miRNAs and cancer development was first reported in chronic lymphocytic leukemia caused by downregulation of miR-15 and miR-16 [81].

Many miRNAs have been reported to be deregulated in GC. MiR-129-2 was silenced in GC and restoration of its expression could trigger apoptosis probably through regulating the relative abundance of proapoptotic and antiapoptotic members of Bcl-2 family [82]. Downregulation of miR-218 in GC is implicated in metastasis resulting from the derepression of its target Robol, a transmembrane receptor for Slit, and thereby enhancing Slit/Robol signaling [83]. The high mobility group A2 (HMGA2) can promote the assembly of regulatory protein complexes at transcriptional sites [84], thus representing as a hallmark of various malignant tumors, including GC. Loss of inhibition by let-7 can contribute to HMGA2 overexpression and enhance transcription in GC tissues [85]. MiR-141, belonging to miR-200 family and reported to inhibit EMT (epithelial-mesenchymal transition) and enhance E-cadherin expression, was implicated reductive obviously in primary GC [86–88]. An additional downregulated miRNA was miR-9 whose target was RAB34 (member RAS oncogene family) [89]. Wan et al. further found miR-9 could inhibit growth by targeting NF-Kb1 (nuclear factor of kappa light polypeptide gene enhancer in B-cells 1) in GC cells [90]. It is also not unusual that miRNAs are overexpressed in GC which is called oncogenic miRNAs. The levels of miR-106a, which belongs to the miR-106b-25 cluster, were significantly higher compared with normal counterparts and also obviously correlated with tumor stage, size, differentiation, and lymphatic and distant metastasis [91]. Therefore, miR-106a might be used as a diagnostic biomarker of GC. Furthermore, two histological subtypes of GC showed different expression pattern of miRNAs. Eight miRNAs such as miR-105 were upregulated in the diffuse type while only four miRNAs such as miR-373 increased in the intestinal type [92]. In clinical practice, these dysregulated miRNAs can be used as different biomarkers in GC for early diagnosis, prognosis, and predictive response to chemotherapy. High levels of miR-17 and miR-106a have been confirmed in a study in which the value of the area under the receiver-operating characteristic curve for combined miR-17/miR-106a assay was 0.741, suggesting miRNAs could be useful biomarkers for early diagnosis of GC [93]. The expression of miR-451 was reduced in GC and related with worse prognosis [94]. By contrast, overexpression of miR-451 leads to reduction of cell proliferation and increase of sensitivity to radiotherapy. These data suggests miR-451 may play a role in suppressing carcinogenesis and could be a target for cancer therapy. There are many miRNAs involved in drug resistance as well. For instance, the overexpression of miR-15b or miR-16 sensitized SGC7901/VCR cells towards Vincristine (VCR) partly via inhibiting Bcl-2 to increase apoptosis [95]. This indicates a potential therapeutic use of miR-15b and miR-16.

In addition to miRNAs in primary and metastatic tumor tissues, cell-free circulating miRNAs can be detected in plasma and serum because these miRNAs are reproducible, consistent, and resistant to RNase [96, 97]. For instance, from a genome-wide miRNA profile approach, miR-378 showed a higher level in serum of GC patients with 87.5% sensitivity and 70.7% specificity [98]. And the differences of miR-378 levels in serum between patients and controls could be detected at early stages of GC. MiR-31 expression is downregulated in GC tissues, and, interestingly, the positive detection rate of the serum miR-31 is much higher than that of the serum CEA (68.29% versus 21.95%). This study indicates that miR-31 may be a novel diagnostic marker for GC [99].

## 4. Epigenetic Biomarkers in Colorectal Cancer and Their Applications

### 4.1. DNA Methylation in CRC

Colorectal cancer (CRC) is also the result of progressive accumulation of genetic and epigenetic alterations in tumor suppressor genes and oncogenes. The former process was first described by Fearon and Vogelstein in a classic adenoma-cancer progression model from which we understand considerably the molecular pathogenesis of CRC [100]. However, the original model provided a relatively limited explanation of molecular alterations. Now, we believe there are different molecular events contributing to the formation of CRC. For example, apart from mutations and other genetic changes, epigenetic silencing of APC through promoter hypermethylation could lead to activation of the Wnt (wingless and integration site growth factor) pathway [101].

There is also increasing evidence that aberrant DNA methylation is an important hallmark of CRC. The link between DNA methylation and CRC was first observed in 1983 when it was suggested that cancer cells occurred because of hypomethylation of their genomes [102]. Genomic instability and loss of imprinting genes like IGF2 (insulin-like growth factor 2) may be both initiated by DNA hypomethylation [103, 104]. Global hypomethylation may influence tumor progression by making chromosomes more susceptible to breakage and cause disruption of normal gene structure and function, leading to reactivating previously silenced retrotransposons [105–107]. A typical example of global hypomethylation is the LINE-1 repeat sequence. LINE-1 hypomethylation has been shown to independently prognosticate poor CRC survival and predict poor response to 5-FU (5-fluorouracil) chemotherapy [108, 109].

Similar to GC, DNA hypermethylation in CpG islands is also postulated to silence the expression of some important genes in CRC. A subset of CRC has a specific phenotype termed as CIMP (CpG island methylator phenotype) with a high proportion of methylated genes promoters [110]. Almost 30%–40% of proximal CRC and 3%–12% of distal CRC are characterized as CIMP [111]. According to epigenetic and clinical profiles, primary CRC is divided into three distinct subclasses: CIMP1, CIMP2, and CIMP negative. CIPM1 has a good prognosis, whereas CIMP2 is associated with poor prognosis [112]. CIMP status of cancers has been assessed as a predictive marker for 5-FU responsiveness [113]. Due to the DNA hypermethylation, some tumor suppressor genes are silenced such as P16, VHL (von Hippel-Lindau tumor suppressor), and MLH1 in CRC [114, 115]. MLH1, a mismatch repair (MMR) gene, is inactivated by promoter methylation, resulting in high-level MSI in some sporadic CRC and then genetic instability to drive tumor onset [116, 117].

In recent years, several DNA methylation markers have been proposed as useful early biomarkers for CRC detection. The detection of aberrant methylation of vimentin in fecal DNA is obvious in CRC when compared with normal control patients [118]. The sensitivity and specificity of methylated vimentin to detect CRC were 88% and 87%, respectively [119]. In addition, the transcription factor GATA4 (GATA binding protein 4) has been identified as a novel biomarker for the detection of CRC with a sensitivity of 51–71% and a specificity of 84–93% based on distinct study groups [120]. Blood-based tests for CRC detection could have the potential for better compliance. The methylation of SEPT9 (septin 9), encoding a GTPase involved in dysfunctional cytoskeletal organization, was detected in the CRC patients with an overall sensitivity of 90% and specificity of 88% [121]. Meanwhile since this marker is not influenced by patients' age, sex, and tumor location, STEPT9 is particularly attractive for biomarker applicability.

Traditional methods cannot sufficiently predict the prognosis of single cancer cases. Clinicians may be not able to accurately decide which patients will be at high risk for recurrence and benefit from chemotherapy. Therefore, it is essential to search for novel biomarkers improving prognosis, and then it would support clinicians in the decision of which patients should receive adjuvant treatment. Promoter methylation of CHFR (checkpoint with forkhead and ring finger domains) was found to be associated with survival and was considered to be an independent predictor for tumor recurrence [122]. Moreover, simultaneous DNA methylation of IGFBP3 (insulin-like growth factor binding protein 3) and CD109 (CD109 molecule) was correlated with worse survival for stage II CRC [123]. The questions of which patients should be treated and why some patients respond to therapy whereas others do not need to be solved as adjuvant cancer therapy imposes unnecessary toxicity and a huge financial burden on patients. Hypermethylation of MGMT (O-6-methylguanine-DNA methyltransferase) has been reported in CRC and inactivation of MGMT was shown to sensitize cells to the effects of alkylating agents [124]. Moving forward, MGMT was able to reduce mutagenic and cytotoxic adducts from guanine in DNA [125]. These data lead to a proposal that MGMT can be used as a predictive marker in CRC. Besides the association with longer survival of CRC patients treated with irinotecan, WRN (Werner syndrome, RecQ helicase-like) hypermethylation appears to be related with mucinous differentiation in CRC [126, 127]. All of these possible markers need to be further validated before they are applied for clinical use.

### 4.2. Histone Modifications in CRC

In addition to alterations in DNA methylation, histone modification patterns also happen in CRC. Despite their various effects, histone modifications have drawn less attention than DNA methylation biomarkers likely due to less predictable transcriptional response and more intensive detection techniques in CRC. The biomarker studies mainly focused on the expression of global histone modifying enzymes. For example, HDAC2 silencing a group of targeting genes has been shown to be independently associated with poor survival in CRC [128]. The most studied histone-associated protein is EZH2 (enhancer of zeste homolog 2), which encodes a H3 methyltransferase to induce polycomb-mediated repression of target genes. EZH2 has shown poor prognostic effects and can promote loss of cellular adhesion and CRC metastasis [129, 130]. Interactions can occur among different histone modification patterns to generate various impacts. Decreased acetylation at H3K9 and increased methylation at H3K9 were associated with silencing of genes such as P16, MLH1, and MGMT. Hypomethylation alone could not reverse silenced genes. Instead augmented histone acetylation with localized hypomethylation allows the turnover of epigenetically silenced genes. After 5-Aza treatment for 10 days, CDO1 (cysteine dioxygenase type 1) was still expressed as it had a localized hypomethylation and an increased histone H3 acetylation [131].

### 4.3. miRNAs in CRC

Similarly, CRC-related miRNAs have also garnered considerable attention as potential biomarkers due to their multifaceted functional roles. MiR-143 and miR-145 are the most extensively studied miRNAs in CRC. They were observed to be downregulated in CRC and ectopic expression of them brought about the inhibition of cell proliferation [132, 133]. Subsequently, their targets have been discovered. K-Ras was identified as a target of miR-143. By inhibiting K-Ras translation, it could suppress CRC growth [134]. More than 50% of CRC cases presented reduced miR-342 and reconstitution of miR-342 induced apoptosis, indicating that miR-342 might act as a proapoptotic gene [135]. The inverse relationship between reduced miR-101 and COX-2 (cyclooxygenase-2) overexpression could confer CRC cells with the ability of growth and invasiveness [136]. P53, the most common mutated tumor suppressor genes, can have an impact on miRNA expression [137]. For example, miR-34a was proved to be regulated directly by P53 and contributed to apoptosis and senescence-like phenotypes via downregulation of the EZF (Kruppel-like factor 4) and SIRT1 (sirtuin 1) and upregulation of P53 and P21 [138–140].

There are also many oncogenetic miRNAs in CRC. For example, miR-21 might function as an oncogene due to its overexpression in CRC [141]. It has been demonstrated that increased expression of miR-21 was correlated with lymph node metastasis and poor survival and response to chemotherapy [142, 143]. These studies suggest that miR-21 could act as a biomarker for gastrointestinal cancers. Of course, there are still discrepancies between GC and CRC. In contrast to the findings that miR-31 expression levels were downregulated in GC, its expression was increased in CRC and correlated with tumor pathological staging, higher expression in stage IV tumors than in stage II tumors. This suggests miRNAs alterations may occur at different stages of colorectal tumorigenesis and malignant progression.

Circulating miRNAs can be detected in plasma and serum of CRC patients as well. Huang et al. showed that miR-29a and miR-92a in plasma discriminated CRC from tissues they arise from with 83.0% sensitivity and 84.7% specificity [144].

## 5. Conclusion and Perspectives

It is well known that various epigenetic alterations, especially aberrant DNA methylation, histone modifications, and miRNAs, are involved in tumorigenesis. Advances in our understanding of the molecular pathology of gastrointestinal cancers by elucidating the relevance of epigenetic alterations might lead to the identification of potential biomarkers for the diagnosis, prognosis, and drug development of GC cancers. With the development of the next generation genome sequencing as well as single molecular PCR (polymerase chain reaction), it became possible to analyze trace amount of nuclear acids, including circulating cell-free DNA, that will be the next promising epigenetic biomarkers for cancer detection. Although some methylated DNAs and miRNAs were found to valuable as a single biomarker for cancer detection, more potential epigenetic biomarkers will be found after the wide application of new sequencing platforms with high speed, depth, and accuracy. Epigenetic signatures, including a panel of methylated DNAs or miRNAs, will show the potential in the early diagnosis or screening and prognosis or therapy response prediction of GI (gastrointestinal) cancers. In addition, such biomarkers could be more sensitive and specific for cancer detection when combined with well-used biochemical biomarkers.
